# Effects of carnosine and histidine-containing dipeptides on biomarkers of inflammation and oxidative stress: a systematic review and meta-analysis

**DOI:** 10.1093/nutrit/nuad150

**Published:** 2023-12-12

**Authors:** Saeede Saadati, Robel Hussen Kabthymer, Giancarlo Aldini, Aya Mousa, Jack Feehan, Barbora de Courten

**Affiliations:** Department of Medicine, School of Clinical Sciences, Faculty of Medicine, Nursing and Health Sciences, Monash University, Clayton, Victoria, Australia; Department of Medicine, School of Clinical Sciences, Faculty of Medicine, Nursing and Health Sciences, Monash University, Clayton, Victoria, Australia; Department of Pharmaceutical Sciences, University of Milan, Milan, Italy; Monash Centre for Health Research and Implementation (MCHRI), School of Public Health and Preventive Medicine, Faculty of Medicine, Nursing and Health Sciences, Monash University, Melbourne, Australia; Institute for Health and Sport, Victoria University, Melbourne, Australia; Department of Medicine, School of Clinical Sciences, Faculty of Medicine, Nursing and Health Sciences, Monash University, Clayton, Victoria, Australia; School of Health and Biomedical Sciences, STEM College, Royal Melbourne Institute of Technology (RMIT) University, Melbourne, Australia

**Keywords:** carnosine, histidine-containing dipeptide, inflammation, meta-analysis, oxidative stress

## Abstract

**Context:**

Carnosine and histidine-containing dipeptides (HCDs) are suggested to have anti-inflammatory and antioxidative benefits, but their effects on circulating adipokines and inflammatory and oxidative stress biomarkers remain unclear.

**Objectives:**

The aim of the present systematic review and meta-analysis was to determine the impact of HCD supplementation on inflammatory and oxidative stress biomarkers.

**Data Sources:**

A systematic search was performed on Medline via Ovid, Scopus, Embase, ISI Web of Science, and the Cochrane Library databases from inception to 25 January 2023.

**Data Extraction:**

Using relevant key words, trials investigating the effects of carnosine/HCD supplementation on markers of inflammation and oxidative stress, including C-reactive protein (CRP), tumor necrosis factor-α (TNF-α), interleukin-6 (IL-6), adiponectin, malondialdehyde (MDA), glutathione (GSH), superoxide dismutase (SOD), total antioxidant capacity (TAC), and catalase (CAT) were identified. Meta‐analyses were conducted using random‐effects models to calculate the weighted mean differences (WMDs) and 95% confidence intervals (CIs).

**Data Analysis:**

A total of 9 trials comprising 350 participants were included in the present meta-analysis. Carnosine/HCD supplementation led to a significant reduction in CRP (WMD: –0.97 mg/L; 95% CI: –1.59, –0.36), TNF-α (WMD: –3.60 pg/mL; 95% CI: –7.03, –0.18), and MDA (WMD: –0.34 μmol/L; 95% CI: –0.56, –0.12) and an elevation in CAT (WMD: 4.48 U/mL; 95% CI: 2.43, 6.53) compared with placebo. In contrast, carnosine/HCD supplementation had no effect on IL-6, adiponectin, GSH, SOD, and TAC levels.

**Conclusion:**

Carnosine/HCD supplementation may reduce inflammatory and oxidative stress biomarkers, and potentially modulate the cardiometabolic risks associated with chronic low-grade inflammation and lipid peroxidation.

**Systematic Review Registration:**

PROSPERO registration no. CRD42017075354.

## INTRODUCTION

Inflammation and oxidative stress are involved in the pathogenesis of a myriad of chronic diseases, including obesity, type 2 diabetes, atherosclerosis and other cardiovascular disorders depression, and chronic liver and kidney disease.^1–5^ Chronic low-grade inflammation is common in obesity, and contributes to endothelial dysfunction and impaired insulin secretion through β-cell dysfunction.^6^^,^^7^ Similarly, oxidative stress, characterized by elevated free radicals and reactive oxygen species (ROS), contributes to insulin resistance, micro- and macrovascular diabetes-related complications, as well as impaired glucose tolerance.^8–11^ These processes are interrelated, with inflammation leading to high oxidative stress in a positive feedback loop.^12^ Moreover, when the levels of oxidative stress are high, endogenous antioxidants, including catalase (CAT), superoxide dismutase (SOD), and glutathione (GSH), may not be able to stop the overproduction of ROS that can harm cellular proteins, lipids, and DNA/RNA, leading to cell death and the development of chronic diseases.^13^

Although anti-inflammatory and antioxidant medications are available, primary prevention methods remain essential to mitigate the growing burden of chronic disease. Previous evidence has demonstrated the anti-inflammatory and antioxidative properties of histidine-containing dipeptides (HCDs) in different chronic conditions.^14–16^ HCDs are a group of soluble peptides and its founding member, carnosine (β‐alanine l-histidine), is either produced naturally in the mammalian heart, skeletal muscle, brain tissue, and kidneys or naturally from food, as well as through dietary supplementation.^14^^,^^17^ Carnosine has been extensively studied in both animal models^18–21^ and human clinical trials^22–27^ in the context of several disease pathologies.

Carnosine could be an effective strategy for ameliorating oxidative stress via its well-established antioxidant activity, which can be attributed to a direct radical scavenging activity and detoxifying effect towards radical and oxidizing species, metal chelating effects,^20^^,^^28^^,^^29^ and as recently found, by activating the expression of members of the endogenous antioxidant system (nuclear factor erythroid 2–related factor 2 [Nrf2] pathway).^30^ Indeed, the administration of 20 mg/100 g of carnosine for 4 weeks was shown to enhance serum levels of glutathione peroxidase (GSH-PX) and superoxide dismutase (SOD) in rats with streptozotocin-induced diabetes.^31^ In addition, carnosine supplementation improved the activities of GSH-PX and SOD and decreased malondialdehyde (MDA) formation and ethanol-induced oxidative damage in Wistar rats,^32^ Sprague–Dawley rats,^33^ and Balb/cA mice.^34^ Carnosine has also been shown to alleviate inflammation through its direct effects on modulating inflammatory cytokine production in mice.^35^^,^^36^

However, data from human studies are notably inconsistent. While some studies support the use of carnosine for reducing markers of oxidative stress and improving antioxidant status,^37^^,^^38^ others suggest that carnosine has no effect on advanced glycation end-products (AGEs) or precursors of advanced lipoxidation end-products (ALEs), such as 4-hydroxynonenal (4-HNE) and MDA.^39^^,^^40^ The effect of carnosine on proinflammatory cytokines is also controversial. One gram per day of carnosine supplementation for 12 weeks resulted in decreased levels of tumor necrosis factor α (TNF-α) with no significant effects on interleukin (IL)-6 (IL-6).^23^ However, 12-week histidine supplementation in women with obesity suppressed inflammation through reduction in TNF-α and IL-6 levels.^41^

The effects of carnosine/HCDs on inflammatory and oxidative stress status have not been previously synthesized. In addition, since the related evidence has had conflicting results, this study aimed to address this knowledge gap by conducting a comprehensive systematic review and meta-analysis of existing randomized controlled trials (RCTs) investigating the effects of carnosine and other HCDs on inflammatory and oxidative stress biomarkers.

## METHODS

The protocol for the present review was developed a priori, preregistered on PROSPERO (CRD42017075354), and published previously.^42^ This review conforms to the updated 2020 Preferred Reporting Items for Systematic Reviews and Meta-Analyses (PRISMA) guidelines.^43^

### Data sources and searches

The electronic databases such as Medline via Ovid, Scopus, Embase, ISI Web of Science, and the Cochrane Library from inception to 25 January 2023 were systematically searched to identify relevant studies. Databases were searched using medical subject headings (MeSH) and non-MeSH terms, which are shown in Table S1 (see the Supporting Information online). No restrictions were applied in terms of language or year of publication. The reference lists of eligible studies were manually searched for the identification of additional studies. Google Scholar was also used to manually search for grey literature (ie, studies not included in scientific databases). The deidentified aggregate data for the purpose of meta-analysis were requested if the necessary data were not reported (maximum of 3 e-mail attempts).

### Study selection

A systematic review management platform (Covidence; Veritas Health Innovation Ltd) was used to import all the titles and abstracts of the papers from the searches. Duplicates were subsequently removed, and the remaining articles were checked for potential eligibility. Two independent reviewers (S.S. and R.H.K.) screened the titles and abstracts of each article found during the initial search. Full texts were then retrieved for all papers that seemed to fulfill the inclusion criteria. Any disagreement regarding the eligibility of the studies was resolved by discussion with a third reviewer (A.M.).

### Eligibility criteria

Studies that met the selection criteria under a predetermined PICOS (Population, Intervention, Comparison, Outcomes, and Study design) framework, as indicated in Table 1, were considered eligible. In brief, the following eligibility criteria were applied—(1) Participants: males or females of any age, ethnicity, medication use, or comorbidities; (2) Intervention: carnosine or related HCDs (anserine, N-acetylcarnosine [NAC], β-alanine, etc), administered alone (pure) and in any form (oral, intravenous, or intramuscular); (3) Comparison: placebo, any pharmacological or nonpharmacological interventions, or usual care; (4) Outcomes: measurement of any inflammatory and oxidative stress outcomes, including CRP, TNF-α, IL-6, adiponectin, MDA, GSH, SOD, TAC, and CAT; and (5) Study design: only RCTs with crossover or parallel designs and systematic reviews of RCTs were included. Systematic reviews were used to find any additional RCTs not captured by the search, which were then located and screened for eligibility.

**Table 1:** PICOS criteria for inclusion of studies

Parameter	Inclusion criteria	Exclusion criteria
Population	Men and women of any age, ethnicity, geographic area, comorbidities, or medication use	Studies not in humans (animal or cell-culture studies)
Intervention	Carnosine and related HCDs (beta-alanine, anserine, NAC, etc) alone (pure) supplementation administered in any form (intravenous, intramuscular, or oral) and of any dosage and for any duration	Studies without carnosine and/or HCD supplementation, or studies that combined carnosine and related HCDs with other supplements (other interventions eg, diet and/or exercise were included if the intervention was delivered in the same way to both groups)
Comparator	Placebo or usual care or any pharmacological or nonpharmacological interventions	Studies with no control group
Outcome	Inflammatory and oxidative stress biomarkers	Studies without outcome of interest
Study design	RCTs in either parallel or cross-over design	Narrative reviews, letters, editorials, non–peer-reviewed studies, conference proceedings

*Abbreviations:* HCD, histidine-containing dipeptide; NAC, N-acetylcarnosine; PICOS, Population, Intervention, Comparison, Outcomes, Study design; RCT, randomized controlled trial.

The following exclusion criteria were applied: (1) animal or cell culture/experimental studies, (2) studies using a combination of carnosine and/or related HCDs (β-alanine, anserine, NAC, etc) with other supplements (other combined interventions such as diet and/or exercise were included as long as the intervention was delivered in the same way to both groups), (3) studies without an appropriate control group, (4) studies not assessing the endpoints of interest, and (5) narrative reviews, non–peer-reviewed literature, conference abstracts, letters, editorials, observational studies, and case reports.

### Data extraction

Two independent reviewers (S.S. and R.H.K.) extracted data from eligible full-text articles using a predefined data extraction form. Extracted data included the following: first author, study location, study design, publication year, sample sizes of the intervention and control groups, dose, frequency, and duration of the intervention, type of supplement, age, health status, and body mass index of the participants, and the study results (mean or median of baseline, follow-up, or difference between baseline and follow-up values [delta], with standard deviations [SDs], 95% confidence intervals [CIs], standard errors [SEs], or interquartile ranges). Data from crossover trials were extracted only for the first phase. All computed data entries and extracted data for the meta-analysis were cross-checked for accuracy.

### Quality assessments

The same independent reviewers assessed the risk of bias in the included studies using the Cochrane Risk of Bias 2.0 tool (RoB 2),^44^ as per the protocol. The randomization and allocation process; the presence of predetermined selection criteria; blinding of participants, investigators, and outcome assessors; dropout rates, statistical power, and analysis methods; outcome assessment and reporting; and author conflicts of interest were all examined as individual quality items. Based on all of these factors, each study received a risk-of-bias grade of either high risk, low risk, or some concerns, and disagreement was resolved by discussion.

The overall certainty of each outcome across included studies was evaluated by 2 independent reviewers (S.S. and A.M.) using the Grading of Recommendations Assessment, Development, and Evaluation (GRADE) approach.^45^ Each outcome was graded as high, moderate, low, or very low based on risk of bias, inconsistency, indirectness, imprecision, and other biases, including publication bias. For the GRADE assessment, risk of bias was assessed using the results of the RoB 2 assessment described above. For inconsistency, visual inspection of forest plots, including the magnitude and direction of effect size estimates, consideration of whether CIs overlapped, and between-study variability were used; these factors were considered in relation to the baseline values and cumulative supplement dose, which could logically explain inconsistency. Variations in the population, intervention, and outcomes of interest were considered for indirectness. Imprecision was rated based on the number of studies for a given outcome and the pooled sample size as well as the width of the CIs.

### Data synthesis and statistical analysis

Stata version 17.0 (StataCorp) was used to analyze data. The mean change and SD of the relevant outcomes were used to calculate the overall effect size, which was presented as weighted mean differences (WMDs) and 95% CIs based on a random-effects model. A random-effects model was chosen as there was both significant statistical and clinical heterogeneity in terms of the study methods and population characteristics between the studies. If the SD change was not reported, the SD change was calculated using the formula provided by the Cochrane Collaboration,^46^ which is as follows: SD = square root [(SD_baseline_)^2^ + (SD_final_)^2^ – (2*R* × SD_baseline_ × SD_final_)], where the correlation coefficient (*R*) = 0.8. In addition, SE was converted to SD using the formula SD = SE × (√n) where SE was reported. The main meta‐analysis pooled all studies using carnosine and related HCDs and reporting inflammatory and oxidative stress markers. Descriptive analysis was used for the studies with inadequate information to be pooled for meta‐analysis. Statistical heterogeneity was assessed using the *I^2^* test, with values more than 40% indicating moderate to high heterogeneity and significance determined by the *P* value for heterogeneity (*P_het_*). Publication bias was evaluated using visual inspection of funnel plot asymmetry and using Egger's regression test.^47^ Sensitivity analyses were conducted where studies with a high risk of bias or having some concerns and studies performed on children were excluded to assess their effects on the overall results. Statistical significance was determined by a 2-tailed *P* value < 0.05.

## RESULTS

### Study selection

**Figure 1:**
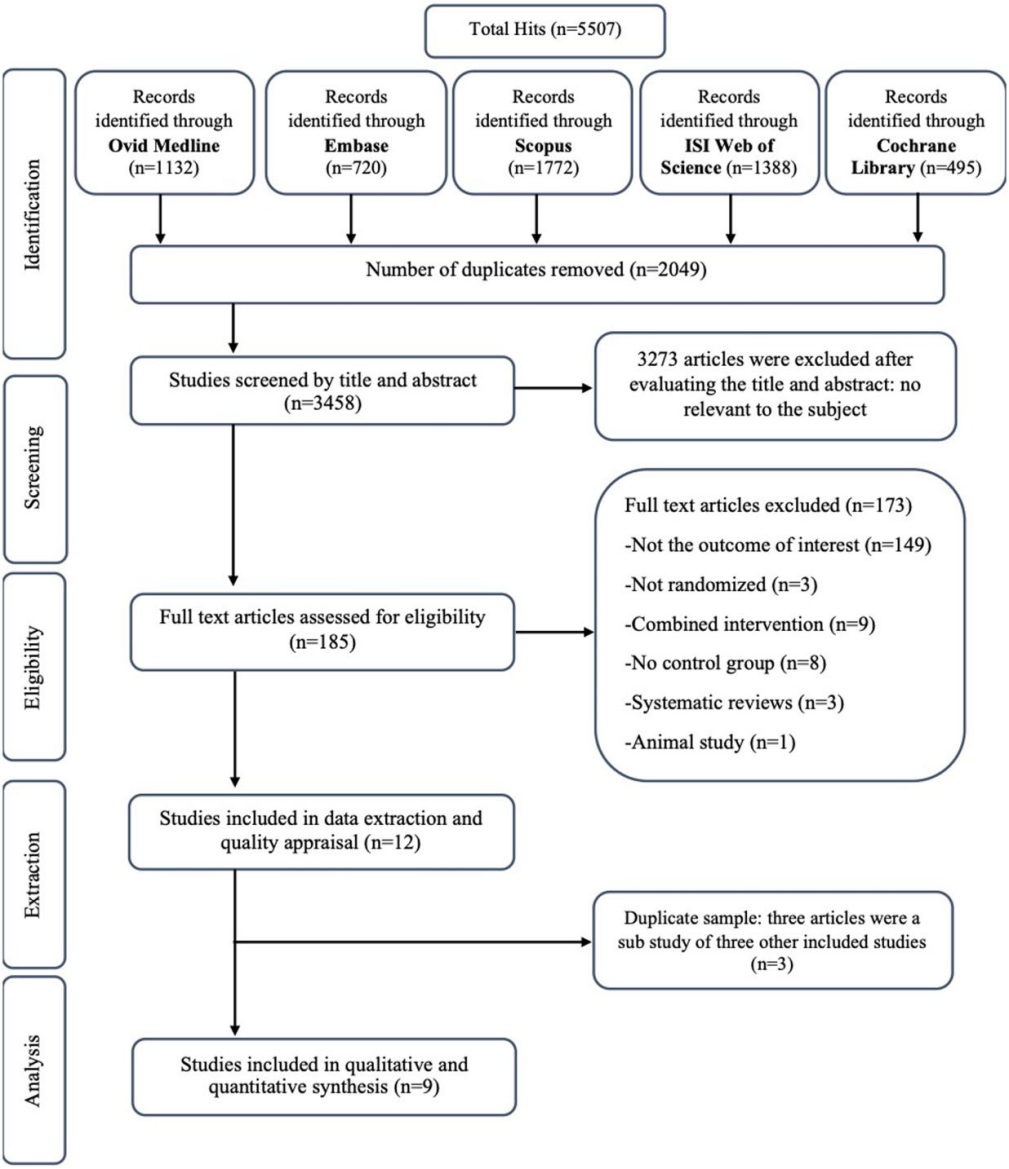
**Consolidated Standards of Reporting Trials (CONSORT) diagram of the screening and selection process for the systematic review and meta‐analysis of the effects of carnosine and histidine-containing dipeptides on inflammatory and oxidative stress biomarkers**.

The process of study selection is depicted in Fig. 1. The primary database search yielded 5507 records. After removing duplicates, 3458 articles remained and were screened by title and abstract, of which 3273 articles were deemed ineligible. The remaining 185 records underwent full-text review. Of these, 173 articles were excluded, due to combined intervention (n = 9); not having a control group (n = 8); failure to report the outcome of interest (n = 149); being an animal study (n = 1); not using a randomized design (n = 3); or being a systematic review (n = 3). The remaining 12 studies proceeded to data extraction; however, 3 studies were removed due to having the same sample of participants; Feng et al^41^ and Du et al,^48^ and references 23,37 and 49,50 used 2 reports from the same studies and hence were treated as a single study. Therefore, a total of 9 unique RCTs were included.

### Study characteristics

The general characteristics of the included studies are described in Table 2.^27^^,^^37^^,^^38^^,^^40^^,^^41^^,^^49^^,^^51–53^ Trials were published between 2013 and 2022 and were carried out in South Korea,^27^ Taiwan,^51^ Iran,^37^^,^^40^ the United States,^52^ Egypt,^38^ Turkey,^53^ Slovakia,^50^ and China.^41^ Of the 9 RCTs, 3^27^^,^^51^^,^^53^ had a crossover design and the rest had a parallel design. Four studies were performed in both genders,^37^^,^^38^^,^^40^^,^^50^ while the rest were conducted only either in males^27^^,^^51–53^ or females.^41^ Two of the 9 studies were in children,^38^^,^^40^ with 1 study in children with autism^40^ and the other in children with type 1 diabetes nephropathy.^38^ The remaining studies were conducted in healthy adults,^27^^,^^51–53^ overweight or obese adults,^41^^,^^50^ and patients with type 2 diabetes.^37^ Participants were excluded if they were taking anti-inflammatory medications^37^^,^^41^ or any other medication that could affect oxidative stress.^50^^,^^51^^,^^53^ However, some participants used antihypertensive medication^38^ and kept their normal dietary intake habit.^40^^,^^52^ One study did not report any information on medication use.^27^ The sample sizes in the studies with adult participants ranged from 5 to 92 participants, and the mean age of participants ranged from 20 to 48 years, whereas in the studies of children, sample sizes ranged from 36 to 85 participants, with a mean age of 8.36 to 13.3 years. Four studies supplemented carnosine alone,^37^^,^^38^^,^^40^^,^^50^ 3 used *β*‐alanine alone,^27^^,^^52^^,^^53^ 1 study used anserine alone,^51^ and 1 study used histidine alone.^41^ Supplementation doses varied from 0.5 g/d to 12 g/d and 1 study used 15 mg/kg and 30 mg/kg as 2 different arms of the same trial.^51^ The duration of supplementation ranged from 1 day to 12 weeks.

**Table 2:** Characteristics of studies included in the systematic review of the effects of carnosine/HCDs on inflammatory and oxidative stress biomarkers

Reference	Country	Design, setting	Participants	Sample size (n) and sex	Sample size, n		Frequency/duration	Mean age, y		Mean BMI, kg/m^2^		Intervention carnosine/HCD-dose control group	Pooled
					IG	CG		IG	CG	IG	CG		
Jin et al (2022)^27^	Korea	Crossover, R, PC, DB	Healthy adults	M (18)	9	9	4 wk	20.78 ± 1.2	20.33 ± 4.36	NR	NR	500 mg/day β-alanine (4 wk) vs placebo	Yes
Alkhatib et al (2020)^51^ (A)(B)^a^	Taiwan	Crossover, R, PC	Healthy adults	M (5)	3	2	24 h	20.9 ± 1.7	20.9 ± 1.7	NR	NR	30 mg/kg anserine vs placebo	Yes
Ghodsi et al (2018)^40^	Iran	Parallel, R, PC, DB	Children with autism	M/F (M: 27; F: 9)	18	18	8 wk	8.92 ± 2.74	8.36 ± 2.90	NR	NR	500 mg/day carnosine vs placebo	Yes
Houjeghani et al (2018)^23^/Houjeghani et al (2018)^37^	Iran	Parallel, R, PC, DB	Adults with T2D	M/F (M: 22; F: 22)	23	21	12 wk	43.0 ± 7.6	40.4 ± 5.1	29.1 ± 5.3	28.3 ± 4.6	1 g/day carnosine vs placebo	Yes
Varanoske et al (2018)^52^	USA	Parallel, R, PC, DB	Healthy adults	M (19)	10	9	2 wk	22.4 ± 3	23 ± 3.8	NR	NR	12 g/day β-alanine vs placebo	Yes
Elbarbary et al (2017)^38^	Egypt	Parallel, R, PC, DB	Children with T1D nephropathy	M/F (M: 43; F: 42)	43	42	12 wk	12.4 ± 3.4	13.3 ± 2.8	NR	NR	1 g/day carnosine vs placebo	Yes
Belviranli et al (2016)^53^	Turkey	Crossover, R, PC, DB	Healthy adults	M (22)	11	11	3 wk	21.7 ± 1.9	21.7 ± 1.9	NR	NR	3.2 g/day β-alanine vs placebo	Yes
Baye et al (2018)^49^/De Courten et al (2016)^50^	Slovakia	Parallel, R, PC, DB	Overweight and obese adults	M/F (M: 18; F: 6)	13	11	12 wk	42 ± 6	43 ± 9	30.4 ± 4.5	32.3 ± 4.6	2 g/day carnosine vs placebo	Yes
Feng et al (2013)^41^/Du et al (2017)^48^	China	Parallel, R, PC, DB	Obese adults with metabolic syndrome	F (92)	47	45	12 wk	45 ± 11	47 ± 10	≥ 28	≥ 28	4 g/day histidine vs placebo	Yes

a A, B was used in multi-arm studies.

*Abbreviations:* BMI, body mass index; F, female; CG, control group; DB, double-blinded; HCD, histidine-containing dipeptide; IG, intervention group; M, male; NR, not reported; PC, placebo-controlled; R, randomized; T1D, type 1 diabetes; T2D, type 2 diabetes.

### Risk-of-bias assessment

**Figure 2:**
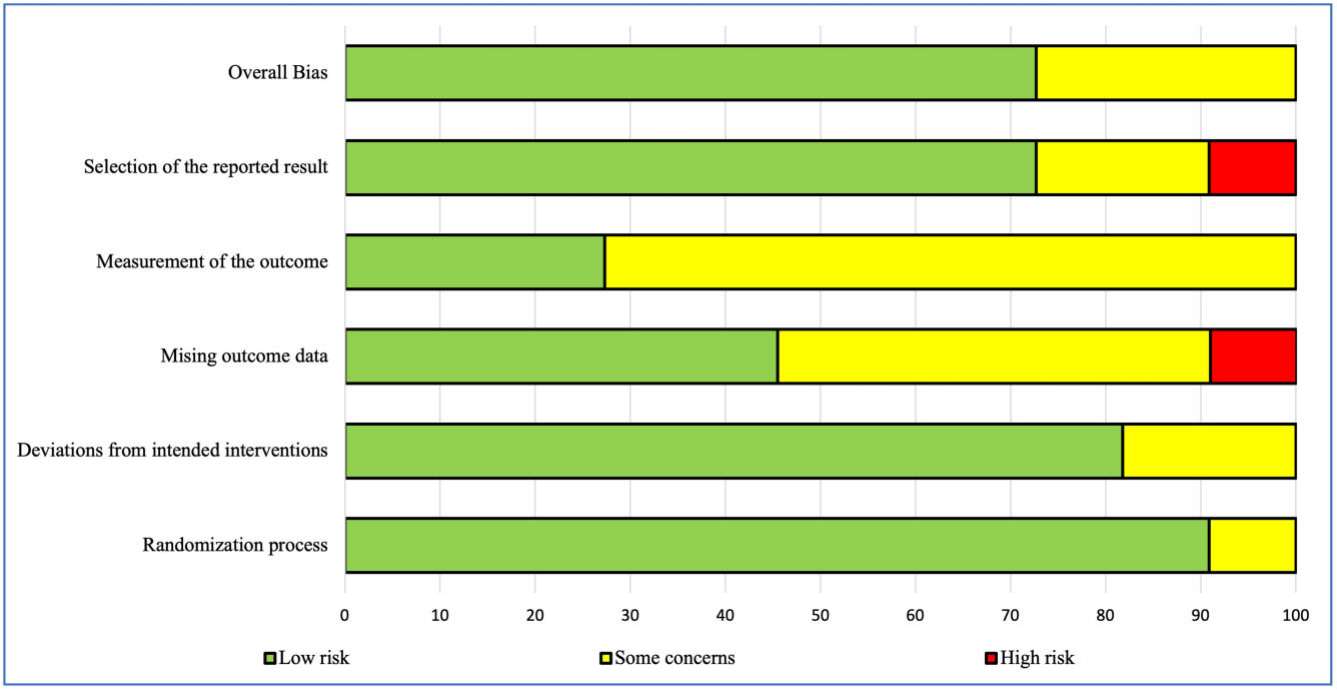
**Risk-of-bias summary**.

Assessments of the methodological quality of the included trials are presented in Fig. 2. Overall, 6 studies were identified as low risk of bias and 3 studies were considered as having some concerns.

### Meta-analysis and sensitivity analysis

All 9 studies were included in the meta-analysis, with a total sample size of 350 participants. The sensitivity analysis also evaluated the effect of each individual study on the total effect size by deleting studies with a high risk of bias or having some concerns and studies performed on children, each study one at a time.

### Effect of carnosine/HCD supplementation on inflammatory cytokines and adipokine

**Figure 3:**
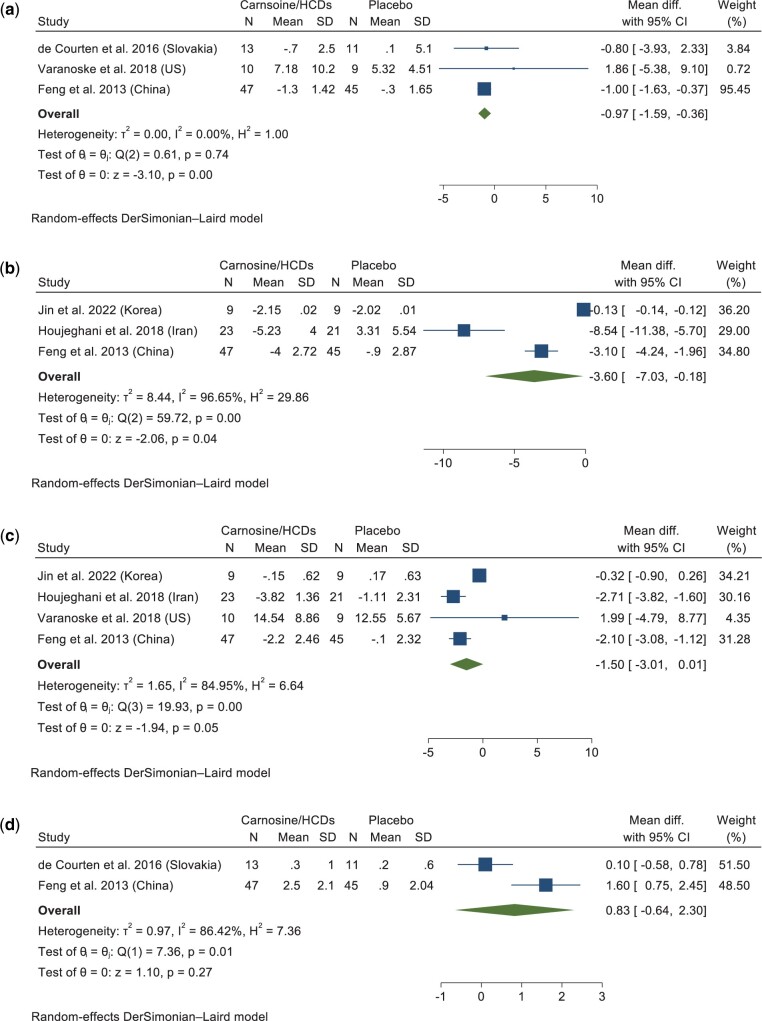
**Forest plot showing results of a meta-analysis of the effects of carnosine/HCD supplementation on a) CRP, b) TNF-α, c) IL-6, d) adiponectin.** Data are reported as weighted mean differences with 95% CIs. *Abbreviations*: CI, confidence interval; CRP, C-reactive protein; diff, difference; HCD, histidine-containing dipeptide; IL-6, interleukin-6; SD, standard deviation; TNF-α, tumor necrosis factor α.

Meta-analysis of data from 3 studies (n = 60 intervention and 52 placebo) demonstrated that carnosine/HCD consumption led to a significant decrease in CRP concentrations (WMD: –0.97 mg/L; 95% CI: –1.59, –0.36; *P* = 0.001; *P_het_* = 0.74, *I^2^* = 0.00%) compared with placebo (Fig. 3a).^41^^,^^50^^,^^52^ All of the studies investigating the effect of carnosine/HCD intake on CRP levels were assessed as having a low risk of bias, except for 1 study,^52^ which was assessed as having some concern of risk of bias. However, when excluding this study in sensitivity analysis, results remained significant.

Pooled data from 3 studies (n = 68 intervention and 63 placebo) indicated that TNF-α levels were reduced significantly in those receiving carnosine/HCDs compared with placebo, although with high statistical heterogeneity (WMD: –3.60 pg/mL; 95% CI: –7.03, –0.18; *P* = 0.03; *P_het_* < 0.001, *I^2^* = 96.65%) (Fig. 3b).^27^^,^^37^^,^^41^ The findings from sensitivity analyses showed that the results were nonsignificant after the exclusion of the study with some concern in terms of risk of bias^41^ (WMD: –4.83 pg/mL; 95% CI: –11.13, 1.47).

There was no significant difference in change in IL-6 between carnosine/HCDs (n = 78) and placebo (n = 72) in a pooled analysis of 4 studies, with a WMD of −1.50 pg/mL (95% CI: –3.01, 0.01; *P* = 0.051) and high heterogeneity (*P_het_* < 0.001, *I^2^* = 84.95%) (Fig. 3c).^27^^,^^37^^,^^41^^,^^52^ Two studies were assessed as having moderate risk of bias,^41^^,^^52^ while the remaining studies were considered as having a low risk of bias. In sensitivity analysis, results were significant after removing the study with some concern in terms of risk of bias^52^ (WMD: −0.98 pg/mL; 95% CI: −1.43, −0.53).

Only 2 studies with a low risk of bias (n = 50 intervention and 43 placebo) reported on adiponectin as an outcome measure. Overall, results demonstrated no difference in adiponectin levels with carnosine/HCD supplementation versus placebo, with high heterogeneity (WMD: 0.83 ng/mL; 95% CI: –0.64, 2.30; *P* = 0.26; *P_het_* = 0.01, *I^2^* = 86.42%) (Fig. 3d).^41^^,^^50^ Sensitivity analysis by risk of bias was not possible due to the small number of studies.

### Effect of carnosine/HCD supplementation on biomarkers of oxidative stress

**Figure 4:**
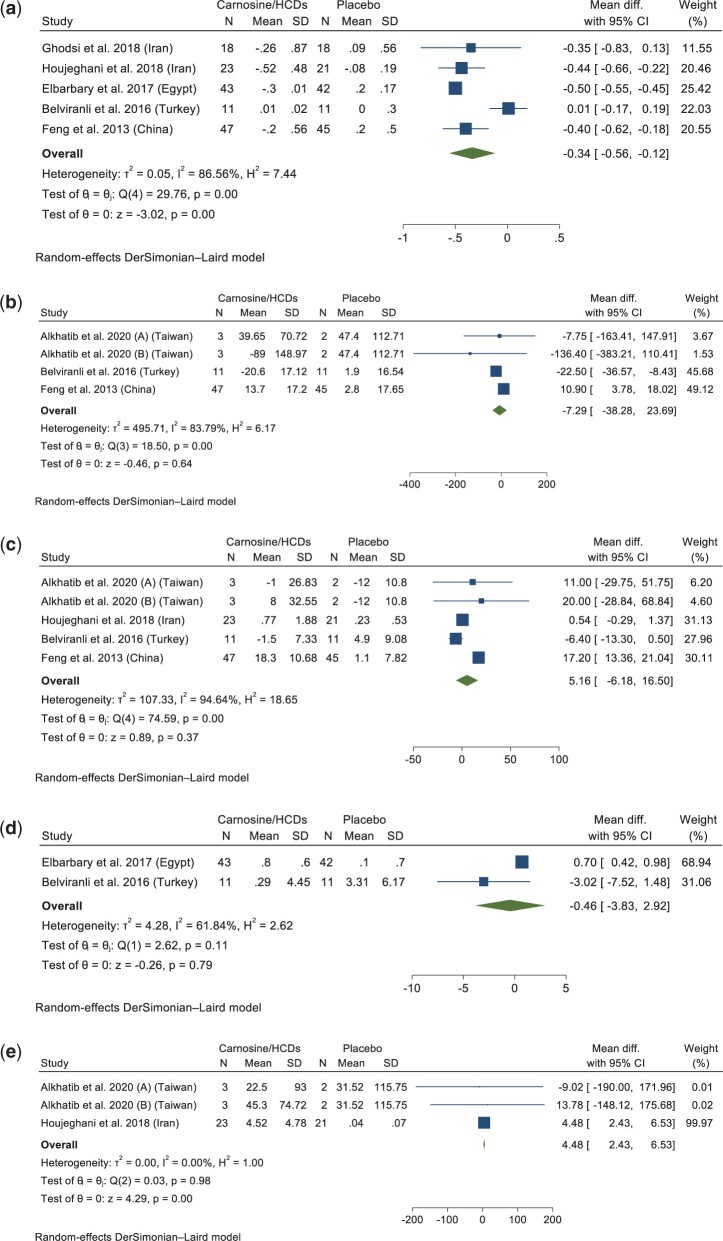
**Forest plot showing results of a meta-analysis of the effects of carnosine/HCD supplementation on a) MDA, b) GSH, c) SOD, d) TAC, and e) CAT.** Data are reported as weighted mean differences with 95% CIs. *Abbreviations*: CAT, catalase; CI, confidence interval; diff., difference; GSH, glutathione; HCD, histidine-containing dipeptide; MDA, malondialdehyde; SD, standard deviation; SOD, superoxide dismutase; TAC, total antioxidant capacity.

A meta-analysis of 5 studies (n = 132 intervention and 124 placebo) showed that carnosine/HCD supplementation significantly decreased MDA levels compared with placebo, with high heterogeneity (WMD: –0.34 μmol/L; 95% CI: –0.56, –0.12; *P* = 0.001; *P_het_* < 0.001, *I^2^* = 86.56%) (Fig. 4a).^37^^,^^38^^,^^40^^,^^41^^,^^53^ All of the studies investigating MDA as their outcome measure were assessed as having a low risk of bias, except for 1 study that had some concern in terms of risk of bias.^41^ In sensitivity analyses, after removing 2 studies (WMD: –1.44 μmol/L; 95% CI: –3.18, 0.29^41^; and WMD: –1.51 μmol/L; 95% CI: –3.06, 0.04^40^), which contributed to the high heterogeneity, the results were nonsignificant.

Pooling of 4 studies (n = 54 intervention and 47 placebo) for GSH showed no significant difference between the carnosine/HCDs and placebo groups (WMD: −7.29 μmol/L; 95% CI: –38.28, 23.69; *P* = 0.64; *P_het_* = 0.00, *I^2^* = 83.79%) (Fig. 4b).^41^^,^^51^^,^^53^ Two studies were assessed to have concerns in terms of risk of bias^41^^,^^51^ and 1 study had a low risk of bias.^53^ According to sensitivity analyses by risk of bias, only excluding the study with some concern^41^ altered the results (WMD: –1.05 μmol/L; 95% CI: –1.81, –0.29). After removing the study^51^ that contributed to heterogeneity, results remained nonsignificant.

There were no significant differences in SOD levels between the carnosine/HCDs and control groups in a pooled analysis of 5 studies (n = 77 intervention and 68 placebo) (WMD: 5.16 U/mL; 95% CI: –6.18, 16.50; *P* = 0.38; *P_het_* = 0.00, *I^2^* = 94.64%) (Fig. 4c).^37^^,^^41^^,^^51^^,^^53^ Except for 2 studies that were assessed to have concerns in terms of risk of bias,^41^^,^^51^ the rest had a low risk of bias. The results remained nonsignificant after the exclusion of the studies with concerns of risk of bias for sensitivity analyses.

Pooled data from 2 studies with a low risk of bias (n = 54 intervention and 53 placebo) indicated that carnosine/HCD supplementation did not change TAC levels compared with placebo (WMD: –0.46 mmol/L; 95% CI: –3.83, 2.92; *P* = 0.79; *P_het_* = 0.11, *I^2^* = 61.84%) (Fig. 4d).^38^^,^^53^ Sensitivity analysis by the risk of bias was not possible due to the limited number of studies.

The effects of carnosine/HCD consumption on CAT levels were evaluated in 3 studies (1 study with a low risk of bias^37^ and 2 studies with some concern in terms of risk of bias [n = 29 intervention and 25 placebo]). Pooled analysis indicated that CAT concentrations increased significantly following carnosine/HCD intake compared with placebo (WMD: 4.48 U/mL; 95% CI: 2.43, 6.53; *P* = 0.00; *P_het_* = 0.98, *I^2^* = 0.00%) (Fig. 4e).^37^^,^^51^ Sensitivity analysis by the risk of bias was not possible due to the small number of studies.

### Publication bias

Based on Egger’s regression test, there was no indication of publication bias for CRP (*P* = 0.50), IL-6 (*P* = 0.17), TAC (*P* = 0.10), or CAT (*P* = 0.98), but there were significant results for TNF-α (*P* < 0.001), adiponectin (*P* = 0.006), MDA (*P* = 0.001), GSH (*P* = 0.03), and SOD (*P* = 0.001), indicating that the results for these may be affected by publication bias. Visual inspection of funnel plots also confirmed these findings (*see*Fig. S1*in the Supporting Information online*).

### GRADE assessment

The GRADE approach was used for the assessment of the certainty of the evidence (*see*Table S2*in the Supporting Information online*). The quality of evidence for CRP was low due to serious indirectness (most of the studies evaluating CRP were conducted in populations with different health statuses) and serious inconsistency (different directions of estimates). For TNF-α, the evidence was of low certainty, downgraded due to serious inconsistency (heterogeneity: *I^2^* = 96.39%, *P_het_* < 0.001) and inclusion of studies with some concern in terms of risk of bias. IL-6 and adiponectin levels were ranked as very low certainty due to serious inconsistency (heterogeneity: *I^2^* = 82.73% and *I^2^* = 86.42%, respectively; *P_het_* < 0.001 and *P_het_* = 0.01, respectively), imprecision (wide CIs), and an indication of inclusion of studies with some concern in terms of risk of bias. For markers of oxidative stress, GSH and SOD were considered as having very-low-certainty evidence, due to serious inconsistency (heterogeneity: *I^2^* = 83.79% and *I^2^* = 94.64%, respectively; both *P_het_* < 0.001), serious imprecision (wide CIs), and indication of inclusion of studies with some concern in terms of risk of bias. Certainty of the evidence for MDA was also very low due to serious inconsistency (heterogeneity: *I^2^* = 86.56%), serious indirectness (analyses included studies that were conducted in children and adults), as well as indication of inclusion of studies with some concern in terms of risk of bias. In addition, the certainty of the evidence for TAC and CAT was also very low due to serious inconsistency (different directions of estimates), serious indirectness (included studies both in healthy adults and individuals with type 2 diabetes), and serious imprecision (small sample size and wide CIs).

## DISCUSSION

This is the first systematic review and meta-analysis to examine the effects of carnosine/HCDs on markers of inflammation and oxidative stress. The findings show that carnosine/HCD consumption significantly reduced CRP, TNF-α, MDA, and CAT levels compared with placebo. No differences were found for other biomarkers assessed, including IL-6, adiponectin, GSH, SOD, and TAC.

The results of this study showed that carnosine/HCD supplementation reduced CRP and TNF-α levels but had no effect on IL-6 and adiponectin levels. Several studies have documented the anti-inflammatory effects of carnosine/HCDs. Recently, a carnosine derivate (resistant to carnosinase) was shown to delay the development of diabetic nephropathy through promotion of renal inflammation in diabetic (*db/db*) mice.^54^ Carnosine also decreased inflammatory molecules, such as CRP and TNF-α levels in Wistar rats,^55^ and reduced apparent nitric oxide (NO) formation, thereby modulating macrophage-mediated inflammatory processes in stimulated murine macrophages.^56^ In addition, 1000 mg/kg per day of carnosine administration for 1 month in rats decreased TNF-α and IL-6 levels.^57^ Apart from the direct effects of carnosine on inflammatory cytokines, carnosine could inhibit AGE and ALE formation and further contribute to the amelioration of inflammation.^58^ No effects on adiponectin were found; however, only 2 studies could be pooled in this analysis. Apart from anti-inflammatory markers of adipose tissue, there is a study showing that 1-week high-dose β-alanine supplementation increases the anti-inflammatory response through elevation of IL-10 concentration during intense military training.^59^ However, it is important to note that there was no study evaluating the effect of carnosine/HCDs on other classic anti-inflammatory markers, such as IL-1 receptor antagonist (IL-1ra). Similarly, only a single study investigated the effectiveness of carnosine supplementation on other adipokines, including serum leptin, resistin, and adipsin,^49^ indicating that carnosine intake reduced serum leptin and resistin levels in adults with overweight or obesity. Despite these divergent results, this review highlights the lack of available data and the need for further research on the impacts of carnosine on adipose tissue inflammation as well as classic anti-inflammatory markers.

Previous in vitro and in vivo studies support the potential antioxidant effects of carnosine. In line with the results from the present study, Aydin et al^60^ demonstrated that 250 mg/kg daily (5 days/wk) carnosine supplementation for 2 months significantly reduced MDA levels in oxidative stress–induced rats. Similarly, 2 other studies in rats reported decreased MDA levels and elevated GSH levels following carnosine supplementation of 1000 mg/kg daily for 1 month and 10 mg/kg twice a week for 1 month.^18^^,^^57^ In vitro, carnosinol, a new carnosine analogue, was shown to increase the activities of SOD and CAT in L6 skeletal muscle cells.^15^ The present data agree with the findings for CAT, showing improved CAT levels following carnosine consumption. Carnosine also increased SOD and GSH levels in carbon tetrachloride–treated human lymphocyte cultures.^61^ However, the findings from this study did not show any difference in GSH and SOD levels in carnosine-treated groups compared with placebo, likely due to the different population groups, various methods for assessment of the endpoints, and a small number of included studies in these analyses.

Numerous mechanisms of action regarding the antioxidant properties of carnosine/HCDs have been proposed. A wide range of antioxidant enzymes are involved in neutralizing ROS, including gamma-glutamyltransferase (GGT), Nrf2, GSH, SOD, CAT, glutathione reductase (GSSG-Rd), GSH-Px, and glutathione *S*-transferase (GST).^62^ Carnosine could increase the expression of the Nrf2 transcription factor, which then leads to upregulation of the vitagenes antioxidant pathway, including heat shock protein 70 (Hsp70), sirtuins (SIRTs), thioredoxin (Trx), gamma-glutamylcysteine synthetase (Gamma-GCs), and heme oxygenase-1 (HO-1).^63^^,^^64^ In Sprague–Dawley rats, intraperitoneal administration of carnosine increased TAC by partial improvement in Trx and GSH/GSSG antioxidant systems and subsequently decreased ROS formation.^65^ Additionally, multiple functions of ROS scavenging^28^^,^^29^^,^^66^^,^^67^ and mitochondrial modulation through preservation of expression of proliferator-activated receptor gamma coactivator-1α (PGC-1α) and sirtuin3 (crucial regulator of mitochondrial function) were attributed to carnosine and its analogues.^15^ Carnosine/HCDs are also proposed to be an effective chelator of heavy metal ions, especially copper and zinc, and consequently aid in detoxification of these toxic elements.^68^

The proposed mechanism for carnosine's anti-inflammatory effect is based on its antioxidant properties. Oxidative stress and inflammation have a close relationship, with each potentially causing the other. Carnosine's antioxidant effects are achieved through direct and indirect mechanisms, with the latter mediated by Nrf2. The ability to activate Nrf2 in oxidative stress conditions or restore its expression is a crucial finding and likely explains many of the benefits associated with carnosine, including its anti-inflammatory action. Furthermore, the fact that carnosine activates Nrf2 independently of electrophilic binding to Keap1 is a significant advantage. Electrophilic Nrf2 activators tend to lack specificity and can affect multiple targets, increasing the risk associated with clinical development.^30^ There are several unanswered questions regarding the mechanism by which carnosine acts as an Nrf2 activator, particularly with regard to whether it can activate Nrf2 in basal conditions or when Nrf2 response is reduced, as seen in certain pathological conditions including inflammation, and further research is needed to understand these mechanisms.

The present study has several strengths. A rigorous search strategy was used and only included RCTs, with no restrictions on the year of publication. To ensure transparency, a predefined protocol for the systematic review was published^42^ and registered on a public forum. Moreover, this meta-analysis encompasses several inflammatory and oxidative stress parameters and adipokines, enabling us to provide a thorough review of the effects of carnosine/HCD supplementation on a range of biomarkers. However, there were limitations in the included studies. The small participant numbers in most of the included studies (all studies had participant numbers <100) was a main limitation in the literature and, as a result, many of the studies were likely underpowered to detect effects on these outcomes. Moreover, there were a limited number of eligible studies for inclusion in the present systematic review and meta-analysis and most of them investigated the effect of carnosine/HCD supplementation for a shorter period. Importantly, the current study also has some limitations. First, subgroup analysis or meta-regression was not performed due to the limited number of included studies. Second, grey literature, non–English-language manuscripts, and non–peer-reviewed papers were not included. Third, these findings might have been impacted by the heterogeneity of the included trials in terms of the populations studied and the supplementation types, doses, and durations. Coupled with the small number of studies, this precluded further sub-investigations, including examining the effects of carnosine in a dose-dependent manner or stratifying different HCD types or population groups. These factors should be considered in future research. Finally, based on GRADE assessment, most of the outcomes of interest were downgraded to low or very low certainty of evidence. As a result, future studies that are well designed and have a rigorous methodology will be helpful in improving the certainty of evidence for the outcomes of interest.

## CONCLUSION

The findings show that carnosine/HCDs may reduce inflammatory biomarkers, including CRP and TNF-α, and oxidative stress markers, such as MDA and CAT, but have no significant effects on IL-6, adiponectin, GSH, SOD, and TAC levels when compared with placebo. However, to strengthen the current body of evidence, larger clinical trials with longer follow-up durations and rigorous methodology to ensure the certainty of evidence of the outcome of interest are necessary.

## Supplementary Material

nuad150_Supplementary_Data

## Supporting Information

The following Supporting Information is available through the online version of this article at the publisher’s website.

*Table S1* **Sample OVID-MEDLINE search strategy**

*Table S2* **GRADE assessment of the effect of carnosine/HCDs on inflammatory and oxidative stress biomarkers meta-analyses**

*Figure S1* **Funnel plots: a) CRP, b) TNF-α, c) IL-6, d) adiponectin, e) MDA, f) GSH, g) SOD, h) TAC, and i) CAT)**

## References

[1] Menzel A , Samouda H, Dohet F, et al Common and novel markers for measuring inflammation and oxidative stress ex vivo in research and clinical practice—which to use regarding disease outcomes? Antioxidants. 2021;10:414. 34071260 10.3390/antiox10060865 PMC8230223

[2] Hussain T , Tan B, Yin Y, et al Oxidative stress and inflammation: what polyphenols can do for us? Oxid Med Cell Longev. 2016;2016:7432797. 27738491 10.1155/2016/7432797 PMC5055983

[3] Minihane AM , Vinoy S, Russell WR, et al Low-grade inflammation, diet composition and health: current research evidence and its translation. Br J Nutr. 2015;114:999–1012. 26228057 10.1017/S0007114515002093 PMC4579563

[4] Caruso G , Fresta CG, Grasso M, et al Inflammation as the common biological link between depression and cardiovascular diseases: can carnosine exert a protective role? Curr Med Chem. 2020;27:1782–1800. 31296155 10.2174/0929867326666190712091515

[5] Hariharan R , Odjidja EN, Scott D, et al The dietary inflammatory index, obesity, type 2 diabetes, and cardiovascular risk factors and diseases. Obes Rev. 2022;23:e13349. 34708499 10.1111/obr.13349

[6] Sun H-J , Wu Z-Y, Nie X-W, et al Role of endothelial dysfunction in cardiovascular diseases: The link between inflammation and hydrogen sulfide. Front Pharmacol. 2019;10:1568. 32038245 10.3389/fphar.2019.01568 PMC6985156

[7] Cieślak M , Wojtczak A, Cieślak M. Role of pro-inflammatory cytokines of pancreatic islets and prospects of elaboration of new methods for the diabetes treatment. Acta Biochim Pol. 2015;62:15–21. 25781159 10.18388/abp.2014_853

[8] Odegaard AO , Jacobs DR, Sanchez OA, et al Oxidative stress, inflammation, endothelial dysfunction and incidence of type 2 diabetes. Cardiovasc Diabetol. 2016;15:51–12. 27013319 10.1186/s12933-016-0369-6 PMC4806507

[9] Lahera V , Goicoechea M, Garcia de Vinuesa S, et al Endothelial dysfunction, oxidative stress and inflammation in atherosclerosis: beneficial effects of statins. Curr Med Chem. 2007;14:243–248. 17266583 10.2174/092986707779313381

[10] Rangel ÉB , Rodrigues CO, de Sá JR. Micro-and macrovascular complications in diabetes mellitus: preclinical and clinical studies. Hindawi 2019;2019:1–5. 10.1155/2019/2161085 PMC6397960 30911551

[11] Ceriello A , Motz E. Is oxidative stress the pathogenic mechanism underlying insulin resistance, diabetes, and cardiovascular disease? The common soil hypothesis revisited. Arterioscler Thromb Vasc Biol. 2004;24:816–823. 14976002 10.1161/01.ATV.0000122852.22604.78

[12] Kim Y-W , West XZ, Byzova TV. Inflammation and oxidative stress in angiogenesis and vascular disease. J Mol Med (Berl). 2013;91:323–328. 23430240 10.1007/s00109-013-1007-3 PMC3656485

[13] García-Sánchez A , Miranda-Díaz AG, Cardona-Muñoz EG. The role of oxidative stress in physiopathology and pharmacological treatment with pro-and antioxidant properties in chronic diseases. Oxid Med Cell Longev. 2020;2020:2082145. 32774665 10.1155/2020/2082145 PMC7396016

[14] Baye E , Ukropcova B, Ukropec J, et al Physiological and therapeutic effects of carnosine on cardiometabolic risk and disease. Amino Acids. 2016;48:1131–1149. 26984320 10.1007/s00726-016-2208-1

[15] Rezzani R , Favero G, Ferroni M, et al A carnosine analog with therapeutic potentials in the treatment of disorders related to oxidative stress. PLoS One. 2019;14:e0215170. 30964920 10.1371/journal.pone.0215170 PMC6456212

[16] Decker E , Livisay S, Zhou S. A re-evaluation of the antioxidant activity of purified carnosine. Biochemistry (Mosc). 2000;65:766–770. 10951093

[17] Kiliś-Pstrusińska K. Carnosine, carnosinase and kidney diseases. Postepy Hig Med Dosw (Online). 2012;66:215–221. 22706107 10.5604/17322693.991600

[18] Ali S , Rasul A, Latif N, et al Effect of levo-carnosine on biomarkers of oxidative stress and hepatotoxicity in cisplatin-treated male Sprague Dawley rats. Pak Armed Forces Med J. 2022;72:1334–1338.

[19] Albrecht T , Schilperoort M, Zhang S, et al Carnosine attenuates the development of both type 2 diabetes and diabetic nephropathy in BTBR ob/ob mice. Sci Rep. 2017;7:44492–44416. 28281693 10.1038/srep44492 PMC5345040

[20] Aldini G , Orioli M, Rossoni G, et al The carbonyl scavenger carnosine ameliorates dyslipidaemia and renal function in Zucker obese rats. J Cell Mol Med. 2011;15:1339–1354. 20518851 10.1111/j.1582-4934.2010.01101.x PMC4373334

[21] Menini S , Iacobini C, Ricci C, et al D‐Carnosine octylester attenuates atherosclerosis and renal disease in ApoE null mice fed a Western diet through reduction of carbonyl stress and inflammation. Br J Pharmacol. 2012;166:1344–1356. 22229552 10.1111/j.1476-5381.2012.01834.x PMC3417451

[22] Derosa G , D'Angelo A, Romano D, et al A clinical trial about a food supplement containing α-lipoic acid on oxidative stress markers in type 2 diabetic patients. Int J Mol Sci. 2016;17:1802. 27801825 10.3390/ijms17111802 PMC5133803

[23] Houjeghani S , Kheirouri S, Faraji E, et al L-Carnosine supplementation attenuated fasting glucose, triglycerides, advanced glycation end products, and tumor necrosis factor–α levels in patients with type 2 diabetes: a double-blind placebo-controlled randomized clinical trial. Nutr Res. 2018;49:96–106. 29420997 10.1016/j.nutres.2017.11.003

[24] Karkabounas S , Papadopoulos N, Anastasiadou C, et al Effects of α-lipoic acid, carnosine, and thiamine supplementation in obese patients with type 2 diabetes mellitus: a randomized, double-blind study. J Med Food. 2018;21:1197–1203. 30311825 10.1089/jmf.2018.0007

[25] Liu Y , Cotillard A, Vatier C, et al A dietary supplement containing cinnamon, chromium and carnosine decreases fasting plasma glucose and increases lean mass in overweight or obese pre-diabetic subjects: a randomized, placebo-controlled trial. PLoS One. 2015;10:e0138646. 26406981 10.1371/journal.pone.0138646 PMC4583280

[26] Yehia R , Saleh S, El Abhar H, et al L-Carnosine protects against oxaliplatin-induced peripheral neuropathy in colorectal cancer patients: a perspective on targeting Nrf-2 and NF-κB pathways. Toxicol Appl Pharmacol. 2019;365:41–50. 30592963 10.1016/j.taap.2018.12.015

[27] Jin S-Y , Moon H-W, Kim J-S, et al Effects of 4 weeks of beta-alanine intake on inflammatory cytokines after 10 km long distance running exercise. Exerc Sci. 2022;31:188–196.

[28] Dursun N , Taşkın E, Öztürk F. Protection against adriamycin-induced cardiomyopathy by carnosine in rats: role of endogenous antioxidants. Biol Trace Elem Res. 2011;143:412–424. 20941549 10.1007/s12011-010-8875-y

[29] Ma J , Xiong JY, Hou WW, et al Protective effect of carnosine on subcortical ischemic vascular dementia in mice. CNS Neurosci Ther. 2012;18:745–753. 22943141 10.1111/j.1755-5949.2012.00362.x PMC6493405

[30] Aldini G , de Courten B, Regazzoni L, et al Understanding the antioxidant and carbonyl sequestering activity of carnosine: direct and indirect mechanisms. Free Radic Res. 2021;55:321–330. 33302741 10.1080/10715762.2020.1856830

[31] AlZahrani I , Badawy A, El-Morshedi N. Antioxidant role of carnosine in type-II diabetic Wistar rats. IJAR. 2014;4:13–17.

[32] Aydın AF , Küskü-Kiraz Z, Doğru-Abbasoğlu S, et al Effect of carnosine against thioacetamide-induced liver cirrhosis in rat. Peptides. 2010;31:67–71. 19958806 10.1016/j.peptides.2009.11.028

[33] Kim MY , Kim EJ, Kim Y-N, et al Effects of α-lipoic acid and L-carnosine supplementation on antioxidant activities and lipid profiles in rats. Nutr Res Pract. 2011;5:421–428. 22125679 10.4162/nrp.2011.5.5.421 PMC3221827

[34] Liu W-h , Liu T-c, Yin M-c Beneficial effects of histidine and carnosine on ethanol-induced chronic liver injury. Food Chem Toxicol. 2008;46:1503–1509. 18222027 10.1016/j.fct.2007.12.013

[35] Tsai S-J , Kuo W-W, Liu W-H, et al Antioxidative and anti-inflammatory protection from carnosine in the striatum of MPTP-treated mice. J Agric Food Chem. 2010;58:11510–11516. 20925384 10.1021/jf103258p

[36] Yan S , Wu S, Yin M, et al Protective effects from carnosine and histidine on acetaminophen‐induced liver injury. J Food Sci. 2009;74:H259–H265. 19799668 10.1111/j.1750-3841.2009.01330.x

[37] Houjeghani S , Kheirouri S, Faraji E, et al Antioxidant status, lipid peroxidation and protein oxidation in type 2 diabetic patients; beneficial effects of supplementation with carnosine: a randomized, double-blind, placebo-controlled trial. Iran Red Crescent Med J. 2018;20:

[38] Elbarbary NS , Ismail EAR, El-Naggar AR, et al The effect of 12 weeks carnosine supplementation on renal functional integrity and oxidative stress in pediatric patients with diabetic nephropathy: a randomized placebo‐controlled trial. Pediatr Diabetes. 2018;19:470–477. 28744992 10.1111/pedi.12564

[39] Saadati S, , Cameron J, , Menon K, et al Carnosine Did Not Affect Vascular and Metabolic Outcomes in Patients with Prediabetes and Type 2 Diabetes: A 14-Week Randomized Controlled Trial. *Nutrients* 2023;15:4835. 10.3390/nu15224835 PMC10674211 38004228

[40] Ghodsi R , Kheirouri S, Nosrati R. Carnosine supplementation does not affect serum concentrations of advanced glycation and precursors of lipoxidation end products in autism: a randomized controlled clinical trial. Ann Clin Biochem. 2019;56:148–154. 30089410 10.1177/0004563218796860

[41] Feng R , Niu Y, Sun X, et al Histidine supplementation improves insulin resistance through suppressed inflammation in obese women with the metabolic syndrome: a randomised controlled trial. Diabetologia 2013;56:985–994. 23361591 10.1007/s00125-013-2839-7

[42] Menon K , Mousa A, de Courten B. Effects of supplementation with carnosine and other histidine-containing dipeptides on chronic disease risk factors and outcomes: protocol for a systematic review of randomised controlled trials. BMJ Open. 2018;8:e020623. 10.1136/bmjopen-2017-020623 PMC5875615 29567852

[43] Page MJ , Moher D, Bossuyt PM, et al PRISMA 2020 explanation and elaboration: updated guidance and exemplars for reporting systematic reviews. BMJ. 2021;372:n160. 33781993 10.1136/bmj.n160 PMC8005925

[44] Sterne JA , Savović J, Page MJ, et al RoB 2: a revised tool for assessing risk of bias in randomised trials. BMJ. 2019;366: L 4898. 10.1136/bmj.l4898 31462531

[45] Guyatt G , Oxman AD, Akl EA, et al GRADE guidelines: 1. Introduction—GRADE evidence profiles and summary of findings tables. J Clin Epidemiol. 2011;64:383–394. 21195583 10.1016/j.jclinepi.2010.04.026

[46] Higgins JP , Thomas J, Chandler J, et al Cochrane Handbook for Systematic Reviews of Interventions. John Wiley & Sons; 2019. 10.1002/14651858.ED000142 PMC10284251 31643080

[47] Egger M , Smith GD, Schneider M, et al Bias in meta-analysis detected by a simple, graphical test. BMJ. 1997;315:629–634. 9310563 10.1136/bmj.315.7109.629 PMC2127453

[48] Du S , Sun S, Liu L, et al Effects of histidine supplementation on global serum and urine 1H NMR-based metabolomics and serum amino acid profiles in obese women from a randomized controlled study. J Proteome Res. 2017;16:2221–2230. 28447460 10.1021/acs.jproteome.7b00030

[49] Baye E , Ukropec J, De Courten MP, et al Carnosine supplementation improves serum resistin concentrations in overweight or obese otherwise healthy adults: a pilot randomized trial. Nutrients. 2018;10:1258. 30205427 10.3390/nu10091258 PMC6165206

[50] De Courten B , Jakubova M, De Courten MP, et al Effects of carnosine supplementation on glucose metabolism: pilot clinical trial. Obesity (Silver Spring). 2016;24:1027–1034. 27040154 10.1002/oby.21434

[51] Alkhatib A , Feng W-H, Huang Y-J, et al Anserine reverses exercise-induced oxidative stress and preserves cellular homeostasis in healthy men. Nutrients. 2020;12:1146. 32325914 10.3390/nu12041146 PMC7231017

[52] Varanoske AN , Wells AJ, Kozlowski GJ, et al Effects of β‐alanine supplementation on physical performance, cognition, endocrine function, and inflammation during a 24 h simulated military operation. Physiol Rep. 2018;6:e13938. 30565426 10.14814/phy2.13938 PMC6299243

[53] Belviranli M , Okudan N, Revan S, et al Repeated supramaximal exercise-induced oxidative stress: effect of β-alanine plus creatine supplementation. Asian J Sports Med. 2016;7:e26843. 27217925 10.5812/asjsm.26843 PMC4870821

[54] Iacobini C , Menini S, Blasetti Fantauzzi C, et al FL‐926‐16, a novel bioavailable carnosinase‐resistant carnosine derivative, prevents onset and stops progression of diabetic nephropathy in db/db mice. Br J Pharmacol. 2018;175:53–66. 29053168 10.1111/bph.14070 PMC5740255

[55] Fadda LM , Attia HA, Al-Rasheed NM, et al Attenuation of DNA damage and mRNA gene expression in hypoxic rats using natural antioxidants. J Biochem Mol Toxicol. 2017;31:e21975. 10.1002/jbt.21975 28833918

[56] Caruso G , Fresta CG, Martinez-Becerra F, et al Carnosine modulates nitric oxide in stimulated murine RAW 264.7 macrophages. Mol Cell Biochem. 2017;431:197–210. 28290048 10.1007/s11010-017-2991-3 PMC5697141

[57] Xie R-x , Li D-w, Liu X-c, et al Carnosine attenuates brain oxidative stress and apoptosis after intracerebral hemorrhage in rats. Neurochem Res. 2017;42:541–551. 27868153 10.1007/s11064-016-2104-9

[58] Stinghen AE , Massy ZA, Vlassara H, et al Uremic toxicity of advanced glycation end products in CKD. J Am Soc Nephrol. 2016;27:354–370. 26311460 10.1681/ASN.2014101047 PMC4731113

[59] Hoffman JR , Gepner Y, Hoffman MW, et al Effect of high-dose, short-duration β-alanine supplementation on circulating IL-10 concentrations during intense military training. J Strength Cond Res. 2018;32:2978–2981. 29746388 10.1519/JSC.0000000000002625

[60] Aydin F , Kalaz EB, Kucukgergin C, et al Carnosine treatment diminished oxidative stress and glycation products in serum and tissues of D-galactose-treated rats. Curr Aging Sci. 2018;11:10–15. 28676006 10.2174/1871530317666170703123519

[61] Alpsoy L , Akcayoglu G, Sahin H. Anti-oxidative and anti-genotoxic effects of carnosine on human lymphocyte culture. Hum Exp Toxicol. 2011;30:1979–1985. 21464095 10.1177/0960327111404908

[62] He L , He T, Farrar S, et al Antioxidants maintain cellular redox homeostasis by elimination of reactive oxygen species. Cell Physiol Biochem. 2017;44:532–553. 29145191 10.1159/000485089

[63] Calabrese V , Scuto M, Salinaro AT, et al Hydrogen sulfide and carnosine: modulation of oxidative stress and inflammation in kidney and brain axis. Antioxidants. 2020;9:1303. 33353117 10.3390/antiox9121303 PMC7767317

[64] Scuto M , Trovato Salinaro A, Modafferi S, et al Carnosine activates cellular stress response in podocytes and reduces glycative and lipoperoxidative stress. Biomedicines. 2020;8:177. 32604897 10.3390/biomedicines8060177 PMC7344982

[65] Milewski K , Hilgier W, Fręśko I, et al Carnosine reduces oxidative stress and reverses attenuation of righting and postural reflexes in rats with thioacetamide-induced liver failure. Neurochem Res. 2016;41:376–384. 26801175 10.1007/s11064-015-1821-9 PMC4773466

[66] Anderson EJ , Vistoli G, Katunga LA, et al A carnosine analog mitigates metabolic disorders of obesity by reducing carbonyl stress. J Clin Invest. 2018;128:5280–5293. 30226473 10.1172/JCI94307 PMC6264636

[67] Cao Y , Xu J, Cui D, et al Protective effect of carnosine on hydrogen peroxide–induced oxidative stress in human kidney tubular epithelial cells. Biochem Biophys Res Commun. 2021;534:576–582. 33276949 10.1016/j.bbrc.2020.11.037

[68] Boldyrev AA , Aldini G, Derave W. Physiology and pathophysiology of carnosine. Physiol Rev. 2013;93:1803–1845. 24137022 10.1152/physrev.00039.2012

