# Supplementary material for: Effects of carnosine and histidine-containing dipeptides on biomarkers of inflammation and oxidative stress: a systematic review and meta-analysis
Source: Nutr Rev. 2023 Dec 12;82(12):1696–709. doi: 10.1093/nutrit/nuad150 (PMC11551452; doi:10.1093/nutrit/nuad150)
Supplement: nuad150_Supplementary_Data [file nuad150_supplementary_data.zip › nuad150_Supplementary_Data/Final_Version_Supplementary Material.docx]

**Table S1.** Sample OVID-MEDLINE search strategy

| 1. Carnosine/ 2. carnosine.mp. 3. beta alanylhistidine.mp. 4. Anserine/ 5. anserine.mp. 6. beta alanyl 3 methylhistidine.mp. 7. ophidine.mp. 8. exp beta-alanine/ 9. beta alanine*.mp. 10. 3 aminopropionic acid.mp. 11. N-Acetyl-Carnosine.mp. 12. N-Acetyl-L-Carnosine.mp. 13. beta alanyl l histidine.mp. 14. beta-ala-his.mp. 15. l histidine beta alanyl.mp. 16. l alpha alanyl l histidine.mp. 17. histidine.mp. 18. balenine.mp. 19. or/1-18 20. randomi?ed controlled trial.pt. 21. controlled clinical trial.pt. 22. randomi?ed.ti,ab. 23. placebo.ti,ab. 24. clinical trials as topic.sh. 25. randomly.ti,ab. 26. trial.ti. 27. or/20-26 28. exp animals/ not exp humans/ 29. 27 not 28 30. Meta-Analysis as Topic/ 31. meta analy$.tw. 32. metaanaly$.tw. 33. Meta-Analysis/ 34. (systematic adj (review$1 or overview$1)).tw. 35. exp Review Literature as Topic/ | 1. or/30-35 2. cochrane.ab. 3. embase.ab. 4. (psychlit or psyclit).ab. 5. (psychinfo or psycinfo).ab. 6. (cinahl or cinhal).ab. 7. science citation index.ab. 8. bids.ab. 9. cancerlit.ab. 10. or/37-44 11. reference list$.ab. 12. bibliograph$.ab. 13. hand-search$.ab. 14. relevant journals.ab. 15. manual search$.ab. 16. or/46-50 17. selection criteria.ab. 18. data extraction.ab. 19. 52 or 53 20. Review/ 21. 54 and 55 22. Comment/ 23. Letter/ 24. Editorial/ 25. animal/ 26. human/ 27. 60 not (60 and 61) 28. or/57-59,62 29. 36 or 45 or 51 or 56 30. 64 not 63 31. 27 or 65 32. 19 and 66 33. limit 67 to humans |
| --- | --- |

**Table S2.** GRADE assessment of the effect of carnosine/HCDs on inflammatory and oxidative stress biomarkers meta-analyses

| **COMPARISON: Carnosine/HCDs *vs.* placebo / control** | | | | | | | | | | | | |
| --- | --- | --- | --- | --- | --- | --- | --- | --- | --- | --- | --- | --- |
|  | **Quality assessment** | | | | | | **No. participants** | |  |  |  |  |
| **No. studies** | **Design** | **Risk of bias** | **Inconsistency** | **Indirectness** | **Imprecision** | **Other** | **Carnosine/HCDs** | **Placebo/ control** | **WMD  [95% CI]** | **Favours** | **Certainty^5^** | **Importance** |
| **Outcome**: CRP | | | | | | | | | | | | |
| 3 | RCT | no serious risk of bias ^1, 5^ | serious^2^ | serious^3^ | no serious imprecision | none | 60 | 52 | -0.97 [-1.59, -0.36] | **Carnosine/HCDs** (lower with carnosine/HCDs ) | ⨁⨁◯◯  LOW | CRITICAL |
| **Outcome**: TNF-α | | | | | | | | | | | | |
| 4 | RCT | serious^1^ | serious^2^ | no serious indirectness | no serious imprecision | none | 68 | 63 | -3.60 [-7.03, -0.18] | **Carnosine/HCDs** (lower with carnosine/HCDs ) | ⨁⨁◯◯  LOW | CRITICAL |
| **Outcome**: IL-6 | | | | | | | | | | | | |
| 3 | RCT | serious^1^ | serious^2^ | no serious indirectness | serious^4^ | none | 78 | 72 | -1.50 [-3.01, 0.01] | No difference | ⨁◯◯◯  VERY LOW | CRITICAL |
| **Outcome**: Adiponectin | | | | | | | | | | | | |
| 2 | RCT | serious^1^ | serious^2^ | no serious indirectness | serious^4^ | none | 50 | 43 | 0.83 [-0.64, 2.30] | No difference | ⨁◯◯◯  VERY LOW | IMPORTANT |
| **Outcome**: MDA | | | | | | | | | | | | |
| 5 | RCT | serious^1^ | serious^2^ | serious^3^ | no serious imprecision | none | 132 | 124 | -0.34 [-0.56, -0.12] | **Carnosine/HCDs** (lower with carnosine/HCDs ) | ⨁◯◯◯  VERY LOW | CRITICAL |
| **Outcome:** GSH | | | | | | | | | | | | |
| 4 | RCT | serious^1^ | serious^2^ | no serious indirectness | serious^4^ | none | 54 | 47 | -7.29 [-38.28, 23.69] | No difference | ⨁◯◯◯  VERY LOW | CRITICAL |
| **Outcome:** SOD | | | | | | | | | | | | |
| 5 | RCT | serious^1^ | serious^2^ | no serious indirectness | serious^4^ | none | 77 | 68 | 5.16 [-6.18, 16.50] | No difference | ⨁◯◯◯  VERY LOW | CRITICAL |
| **Outcome:** TAC | | | | | | | | | | | | |
| 2 | RCT | no serious risk of bias | serious^2^ | serious^3^ | serious^4^ | none | 54 | 53 | -0.46 [-3.83, 2.92] | No difference | ⨁◯◯◯  VERY LOW | CRITICAL |
| **Outcome** CAT | | | | | | | | | | | | |
| 3 | RCT | no serious risk of bias | serious^2^ | serious^3^ | serious^4^ | none | 29 | 25 | 4.48 [2.43, 6.53] | **Carnosine/HCDs** (Higher with carnosine/HCDs ) | ⨁◯◯◯  VERY LOW | CRITICAL |

^1^ Downgraded once due to high or moderate risk of bias for some studies or outcomes

^2^ Downgraded once for inconsistency due to variations in effect estimate directions and/or CIs, or heterogeneity as determined by the *I^2^* statistic

^3^ Downgraded once for indirectness due to the use of different criteria and/or tools/ methods across included studies

^4^ Downgraded once for imprecision due to not having significant effect on IL-6, adiponectin, GSH, SOD, TAC, and CAT (CI 95% includes 0)

^5^ Downgraded once for including high risk studies, then upgraded once since the effect persisted in sensitivity analysis which included only the low risk of bias studies

^6^ ⊕ = serious limitation; ◯ = no serious limitation

a)

b)

c)

**Figure S1.** Funnel plots of a) CRP, b) TNF-α, c) IL-6 with more than two studies for identification of publication bias

d)

e)

f)

**Figure S1 (cont.).** Funnel plots of d) adiponectin, e) MDA, f) GSH with more than two studies for identification of publication bias

g)

h)

i)

**Figure S1 (cont.).** Funnel plots of g) SOD, h) TAC, i) CAT with more than two studies for identification of publication bias
